# British Institutions for the Care of the Inebriate.—VII

**Published:** 1904-01-09

**Authors:** 


### HOSPITAL ADMINISTRATION.

### CONSTRUCTION AND ECONOMICS.

### BRITISH INSTITUTIONS FOR THE CARE OF THE INEBRIATE.—VII.

BY OUR SPECIAL MEDICAL COMMISSIONER.

*Concluded from page* 249.

THE DUXHURST INDUSTRIAL FARM COLONY, REIGATE.

IN the treatment of inebriety mediæval views still persist and primitive methods widely prevail. It is difficult to shake off the heavy fetters riveted by a false and ancient mental attitude, and it is no easy task to depart from the narrow way marked out by ignorance and hedged in by prejudice. There is much remaining of the barbaric in our procedures for dealing with the inebriate subject, and not a little of our difficulty in arresting alcoholism arises from erroneous conceptions regarding its pathology.

Recent researches have thrown a flood of light on some of the most intricate problems concerning disease, and have indicated means not only for securing prophylaxis, but in many instances measures whereby restoration to health may be attained.

The modern spirit of scientific investigation has led to a serious study of the physical and psychological aspects of inebriety, and many of the perplexities which have for so long enveloped this subject with intricate entanglements have been dissipated. Knowledge regarding the pathology of inebriety is being placed on a secure scientific foundation; and already treatment is becoming more rational and consequently more successful. It is, however, much to be regretted that the care of the inebriate in only too many instances is still undertaken by those who are in no ways imbued with the scientific spirit, and are also to a great extent fossilized by a stultifying obedience to an old time empiricism. It cannot be denied that many institutions claiming to administer to the needs of the inebriate are conducted primarily for selfish purposes, and dominated by financial considerations rather than guided by scientific and philanthropic reasons. Reform in the conduct of a considerable number of the so-called inebriate retreats in this country is urgently called for.

With the sweeping away of misconceptions concerning the nature of inebriety, born of superstition and nursed by tradition, it has been possible to secure, at least in some measure, an adoption of rational methods of treatment. Careful clinical investigation of the physical states underlying alcoholism, and a patient study of the psychology of the inebriate is rapidly revolutionising treatment, even as open-air methods by their success in the arrest of consumption are banishing the long prevalent and hopeless measures of restraint under what we now know were most insanitary conditions; so the benefits resulting from the adoption of hygienic methods in the restoration of the inebriate may be expected to overturn speedily the irrational procedures of coercion and constraint prevailing to a greater or less extent in many institutions.

In the overthrow of the morbid, and in a restoration to the normal, a recognition of hygienic laws must be allotted the first place, and in the successful combat with inebriety chief reliance must be placed in the rational application of natural forces.

The Duxhurst Industrial Farm Colony at Reigate, of which Lady Henry Somerset, the founder, is Honorary Superintendent, exemplifies in a striking manner the scientific application of what we have ventured to call the hygienic treatment of inebriety. The institution is intended only for female cases. There is an influential Advisory Board, numerous patrons, a warden, chaplain, and experienced staff, including a medical officer, who, however, is non-resident.

The Colony has now been in existence for upwards of ten years. It is situated in a particularly charming district of Surrey four miles from Reigate and three and a half from Horley, and lies off the old coach road from London to Brighton. The estate consists of some 200 acres.

The settlement may be roughly divided into two parts: the old Manor House, where the well-to-do cases have their quarters, and the cottages where the working class patients reside. The main portion of the village, if such it may be called, consists of cottages built round the three sides of "a green." Each cottage is one-storied, of artistic design, peculiarly attractive with its thatched roof and rose-covered walls, and having numerous well-designed windows, and withal presenting an air of homely simplicity, peace, and comfort. The cottages vary in size, each accommodating from four to eight patients under the charge of a Sister. In each cottage there is a general sitting-room, Sister's quarters, and simple kitchen. The patients, for the most part, have separate apartments, but there are a few larger double rooms.

Every cottage has its distinctive name, and throughout there is evidence of the careful cultivation of an environment making for the development of a true spirit of domestic refinement. One cottage, larger than the rest, is composed of two long apartments, the dining-room and work-room. The former is an excellently-designed apartment, capable of seating about 60 persons; it is thoroughly ventilated, admirably lit, and conspicuous for its cleanliness and comfort.

One of the most attractive and important centres in the colony is the work-room, which indeed is altogether different from institutional apartments generally so termed. The Duxhurst work-room at once strikes the visitor by its quaint artistic beauty, with its hand-looms, spinning-wheels, and tables strewn with interesting textiles.

Here on the occasion of our visit we found a number of patients under the tuition of a special Sister engaged in rug-making, weaving, embroidery, and other work, all of which by its attractive beauty and interesting technique may be expected to exert a beneficial influence in the mental and moral training of these psychologically deranged subjects. It was difficult to understand that the delicate embroidery and carefully executed linen work submitted to us for inspection were the product not only of unskilled workers, but the outcome of the labour of hands and brains that within a few months had been extensively impaired by toxic influences.

While each cottage manages its own laundry duties we were glad to find that patients were not set to the stultifying work of long hours at the wash-tub. The monotony of long periods of plain sewing is also wisely avoided, and wholesome exercise is insisted upon.

Much importance is rightly directed to open-air pursuits.

The patients work in the garden and forcing-houses, help in fruit picking and packing, assist in the care of bees, or in connection with poultry and other suitable farm avocations. They are also taught seed-sorting, basket-making and the like. The out-door work is conducted under the direction of a fully-trained lady gardener.

A particularly interesting feature of the Duxhurst Colony is "The Nest"—a permanent home for children rescued from vicious homes and intemperance. The presence of these children, all involuntary sufferers from the evils necessarily attending inebriety, has, as might be expected, a markedly beneficial influence on the inebriate patients. The children, whose ages range from two to fourteen, are under the charge of a motherly Sister and spend their mornings happily in the village school, coming home late in the day to their own bright nursery and playgrounds. Suitable employments are found for them when they leave "The Nest."

There is a beautiful little church decorated by work modelled by Lady Henry Somerset, and fitted with a handsome carved wood screen contributed by former patients.

From this necessarily brief description it will be evident that the management of the cases is directed not only by sound philanthropic sentiment but by a clear recognition of the special needs of the pathological condition of the patients. Throughout the colony the right hospital spirit prevails. The colonists are treated as patients, and those having the responsibility of their care appear to be imbued with a spirit born of clinical experience, thorough training, and natural ability for exercising directing power over the mind and body of the human being. Every effort is made to break the vicious continuity in the lives of the patients. Strict discipline is maintained, but the institutional hand is encased in the sympathetic and artistic glove. The open-air life makes for general invigoration, and a wisely-directed engagement in suitable employment is wholesome to mind and body. The environment is beneficial to the whole being. The atmosphere of the Colony makes for the evolution of altruistic tendencies, and its spirit is restorative.

All the cases are under strict medical supervision. Dr. A. R. Walters, of Reigate, is the medical officer. Careful records are kept of every patient. On admission, a thorough medical examination is made, and throughout the period of residence a watchful oversight is maintained. Treatment is based on sound rational lines, and conducted in accordance with scientific methods, free from all fads and fancies.

Results, as far as we can ascertain, have been particularly encouraging. Dr. A. R. Walters, in an appendix to the 1902 issue of Dr. Welsh Branthwaite's official report as Government Inspector of Retreats, gives an analysis of 241 cases dealt with from 1896 to 1901, from which it would appear that 59·13 per cent. of the cases have been dealt with successfully. We understand that the results obtained during recent years have been still more encouraging, and "cures" are now returned at over 65 per cent.

Some little time since two cottages were set aside for the reception of "committed" cases, but as might have been expected the association of such with "voluntary" patients proved detrimental to the general welfare of the colony.

The institution is licensed under the 1879 Act for the reception of persons voluntarily applying for admission. Accommodation can be provided on the colony for from 70 to 80 cases, but we understand that something like 3,000 applications have to be refused each year. A report containing full particulars regarding expenditure and receipts and the general work of the colony is published annually. The institution is by no means self-supporting, although all adult patients contribute towards their maintenance. Donations and subscriptions are solicited, and we learn that £300 will build and equip a cottage, and £10 will furnish a room.

We commend the Duxhurst Industrial Farm Colony to the consideration of all interested in the restoration of the female inebriate, believing as we do that it has been designed and is being conducted in accordance with natural methods rich in curative influences.

